# Obturator Hernia: A Rare Cause of Small Bowel Obstruction

**DOI:** 10.4274/balkanmedj.2018.0045

**Published:** 2018-11-15

**Authors:** Long-Zhi Zheng, Wei Lin, Jian Guo, Si-Zeng Chen

**Affiliations:** 1 Department of Gastrointestinal Surgery, The Affiliated Hospital of Putian University, Putian, China; 2 Department of Gastrointestinal Surgery, The First Affiliated Hospital of Fujian Medical University, Fuzhou, China

An 85-year-old female was admitted to our hospital on May 6, 2017 because of abdominal pain since 4 days. She gave an account of the unexplained 96-h history of colicky abdominal pain, vomiting, abdominal distension, and constipation. She experienced a bout of vomiting and more pain around the umbilicus, which continued with relief intervals. She vomited feculent contents of the small intestines, well-colored with bile and appearing more red than natural. In addition, during the last 5 years, she was experienced random bouts of pain across the bowels, with sickness and more or less constipation, from which she was relieved by mild aperients, injections, etc. Upon presentation, she was slightly dehydrated and frail, thin senior woman (height, 150 cm; weight, 32 kg; body mass index, 14.22 kg/m^2^). Her abdomen was distended and generally tender, but no masses were palpable. Bowel sounds were hyperactive, no hernia was perceptible, and results of rectal examinations were negative. Furthermore, preoperative computed tomography revealed a right obturator hernia (Figure 1).

Nonspecific symptoms and signs rendered preoperative diagnosis difficult, which correlated with high mortality in an obturator hernia reported as high as 40% (1). Computed tomography is the gold standard of preoperative diagnosis (2). Thus, once an obturator hernia is diagnosed, it should be surgically treated. Accordingly, we treated the patient surgically after obtaining informed consent from a direct relative. As the poor general condition of the patient demanded speedy completion of the operation, we made a lower midline abdominal incision. The intraoperative exploration revealed pelvic effusion and right obturator hernia with ileal strangulation (Richter’s hernia) (3). We dilated the constriction using a vascular clamp and reduced the hernia. Application of hot towels to the black spot for a few minutes completely restored blood circulation, and as the mesenteric vessels were never compressed, the risk of subsequent gangrene was not considered. Thus, we placed the bowel back into the abdomen. The hernial canal was ~2 cm long, with the uniform caliber and just about the tip of the little finger. The defect was small, but as the patient’s condition had deteriorated at that time, we sutured the edges together to close the defect with a nonabsorbable suture. The postoperative course was uneventful, and the patient was discharged on the postoperative day 7. At the 3 month follow-up, she was well and had regained some of the weight that she had lost.

## Figures and Tables

**Figure 1:**
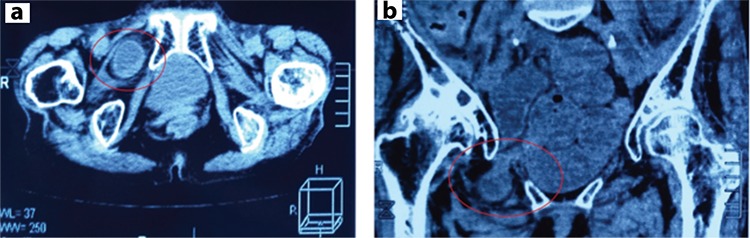
a, b. Computed tomography showing a right obturator hernia (a), cross-section and (b) coronal section-showing a bowel segment with hydro-aerial levels through the right obturator canal (circles).
